# Over-expression of CRTH2 indicates eosinophilic inflammation and poor prognosis in recurrent nasal polyps

**DOI:** 10.3389/fimmu.2022.1046426

**Published:** 2022-11-18

**Authors:** Wenhui Chen, Shaojuan He, Xinyu Xie, Xiaorong Yang, Chen Duan, Ping Ye, Xuezhong Li, Monica G. Lawrence, Larry Borish, Xin Feng

**Affiliations:** ^1^ Department of Otorhinolaryngology, Qilu Hospital of Shandong University, National Health Commission (NHC) Key Laboratory of Otorhinolaryngology, Shandong University, Jinan, Shandong, China; ^2^ Department of Otolaryngology-Head and Neck Surgery, Beijing Tongren Hospital, Capital Medical University, Beijing, China; ^3^ Clinical Epidemiology Unit, Qilu Hospital of Shandong University, Jinan, Shandong, China; ^4^ Department of Medicine, University of Virginia Health System, Charlottesville, VA, United States; ^5^ Department of Microbiology, University of Virginia Health System, Charlottesville, VA, United States

**Keywords:** chemoattractant receptor-homologous molecule expressed on Th2 cells (CRTh2), chronic rhinosinusitis with nasal polyps, eosinophils, prognosis, recurrent nasal polyp

## Abstract

**Background:**

Chronic rhinosinusitis with nasal polyps (CRSwNP) is often characterized by recurrent nasal polyp (NP) growth following surgical removal, but the mechanisms are still not clear. This study aimed to investigate the expression of chemoattractant receptor-homologous molecule expressed on Th2 cells (CRTH2) receptor on NP and the role it plays in eosinophil inflammation and polyp recurrence.

**Methods:**

Forty-one CRSwNPs patients and seventeen controls were enrolled in this study. mRNA was extracted from nasal tissues and evaluated for expression of CRTH2. Immunofluorescence staining was performed to confirm the distribution and expression of CRTH2 protein. CRTH2 expression on peripheral blood eosinophils was quantified by flow cytometry. The eosinophil count and clinical implications were also evaluated and their correlations with CRTH2 expression were analyzed.

**Results:**

Nasal polyps displayed increased expression of CRTH2 in mRNA level compared with control samples, with the highest expression observed in recurrent NP. Immunofluorescence confirmed over-expression of CRTH2 in recurrent NP and this was independent of the concurrent presence of asthma. CRTH2 expression was positively correlated with tissue eosinophil number (Spearman’s ρ=0.69, *P*<0.001) and the postoperative sino-nasal outcome test-22 (SNOT-22) score (Spearman’s ρ=0.67, *P*<0.001). Receiver operating characteristic (ROC) curves revealed CRTH2 was more predictive for NP recurrence compared to either eosinophil number and concomitant asthma, with an area under the ROC curve of 0.9107.

**Conclusion:**

The over-expression of CRTH2 in recurrent nasal polyps correlates with greater eosinophilic inflammation and poor prognosis which is independent of concomitant asthma.

## Introduction

Chronic rhinosinusitis (CRS) is divided into CRS with NP (CRSwNP) and CRS without NP (CRSsNP) (1) by the presence or absence of nasal polyps. A common feature of CRSwNP is the frequent recurrence of nasal polyps following surgical removal, especially for eosinophilic nasal polyps (2, 3), for which current therapies often prove inadequate (1, 4). Although studies have demonstrated eosinophilia is associated with the recurrence of nasal polyps (5–8), elimination of polyp eosinophils did not decrease nasal polyp size and symptoms in a clinical trial (9), suggesting, beyond eosinophils, there are still other mechanisms responsible for NP recurrence. Therefore, further explorations of the factors contributing to eosinophilic inflammation and nasal polyp recurrence are important for the treatment of CRSwNP.

Prostaglandin D_2_ (PGD_2_) is generally considered to be released by mast cells (10, 11), type 2 helper T lymphocytes (Th2) (12), macrophages (13), and eosinophils (14). It contributes to chemotaxis and activation of Th2 cells, eosinophils, basophils, and group 2 innate lymphoid cells (ILC2) through its receptor chemoattractant receptor–homologous molecule expressed on Th2 lymphocytes (CRTH2/DP2), and thus plays an important pro-inflammatory role in asthma and allergic diseases. Studies have shown that CRTH2 is up-regulated in patients with asthma and allergic rhinitis (15, 16), and CRTH2 receptor antagonists can reduce the antigen-induced increase in nasal airway resistance and local eosinophil infiltration in sensitized mice (17). In clinical trials, CRTH2 antagonists have been reported to be effective in the treatment of asthma and allergic diseases (18–20), especially in those with more eosinophils, but recent studies with higher level of evidence did not show a statistically significant improvement in the treatment of asthma (21–23), thus the clinical benefits of CRTH2 antagonists remained to be confirmed. However, these latter studies did not specifically recruit subjects likely to display high levels of CRTH2. Given the heterogenous responses to CRTH2 antagonists, there is a need to identify the most responsive disease endotype likely to respond to CRTH2 antagonism.

At present, the expression and the role that CRTH2 plays in CRSwNP is still controversial (24, 25), especially in those with recurrent nasal polyps, has not been reported. Our previous study (14) demonstrated over-expression of PGD_2_ and hematopoietic prostaglandin D_2_ synthase (hPGDS) in eosinophils of patients with aspirin-exacerbated respiratory disease (AERD), a condition that is characterized by recurrent nasal polyps (rNP), but the expression of CRTH2 receptors was not examined. Here, we further hypothesized that CRTH2 would be highly expressed in patients with rNP, which would thereby enhance the eosinophilic inflammation and contribute to the poor prognosis of CRSwNP. The current studies aimed to (i) investigate whether CRTH2 is highly expressed in patients with rNP and examine its distribution, (ii) analyze the association of CRTH2 expression with eosinophilic inflammation and prognosis of CRSwNP, and (iii) further explore the clinical implications of CRTH2.

## Methods

### Patient recruitment

This study was case-control designed. Subjects who satisfied the diagnostic criteria for CRSwNP established by European Position Paper on Rhinosinusitis and Nasal Polyps (EPOS2012) (26) and subjects with deviated nasal septum were recruited from patients referred to the Department of Otolaryngology, Qilu hospital of Shandong University for endoscopic nasal surgery from January 2018 to October 2020. Excluded were subjects with fungal sinusitis, cystic fibrosis, immunodeficiency diseases, or tumors. Patients treated with a systemic glucocorticoid within 4 weeks prior to recruitment were also excluded as glucocorticosteroids may theoretically influence mediator release. Finally, 41 CRSwNPs and 17 patients with deviated nasal septum (control group) were included. The postoperative therapeutic regimens included daily nasal saline irrigation, mometasone furoate spray 200 μg once daily for 12 weeks and endoscopic debridement. All of the CRSwNP patients were followed postoperatively for more than 12 months *via* endoscopy. For the patients who were found to have recurrent nasal polyps during follow-up, postoperative methylprednisolone 24 mg daily × 14 days was applied. This study was approved by The Medical Ethics Committee of Qilu Hospital of Shandong University and all patients provided written informed consent before enrollment.

The non-recurrent nasal polyp group (Non-rNP) was defined as patients whose NP did not recur during the subsequent 12 months postoperatively. The CRSwNP subjects whose NP recurred according to CRSwNP diagnostic criteria (26) within the 12 months that followed after surgery were classified as recurrent nasal polyp group (rNP) (27). Concomitant asthma was diagnosed according to the Global Initiative for Asthma (GINA) 2014 (28) criteria and confirmed by a respiratory specialist in our hospital.

### Clinical data and sample collection

Nasal polyps of patients with CRSwNP and middle turbinate mucosa of patients with deviated nasal septum were collected intraoperatively. All the tissues were divided into two portions, and one was fixed in a 10% neutral formaldehyde solution to embed in paraffin for immunohistochemical analysis, while the other was immediately snap-frozen in liquid nitrogen and stored at -80°C until processed. Due to the size of tissue samples, not all experiments were done on all participants. Blood samples were obtained to determine the circulating absolute eosinophil count before surgery. Each patient underwent a high-resolution CT scan of the paranasal sinuses and scored preoperatively with the Lund-Mackay scoring system (range: 0–24) (29) by an experienced physician. The SNOT-22 Questionnaire (30, 31) were assessed preoperatively and 12 months postoperatively.

### RNA extraction and qRT-PCR

Quantitative real-time PCR was used to quantify mRNA levels of hPGDS and CRTH2. Total RNA was extracted using Trizol Reagent (Sigma-Aldrich Co., St. Louis, MO, USA) from nasal tissues according to the manufacturer’s protocols. Concentration and RNA quality were determined using the Nanodrop 2000 Spectrophotometer (Thermo Fischer Scientific, USA). Reverse transcription was performed with 1μg of RNA in a 20 μl reaction using TaqMan Reverse Transcription kit (Vazyme Biotech Co., Ltd, China). Reactions went through 15 min at 50°C, 5 min at 85°C in a Bio-Rad thermocycler. PCR reactions contained 15 ng of cDNA (total RNA equivalent) in 5ul HiScript III RT SuperMix for qPCR and primers. The assay was performed using the ABI 7900HT Fast Real-time PCR system (Applied Biosystems, USA). The primers for CRTH2 were as follows: sense, 5’-TGCAAACTGCACTCCTCCAT-3’, antisense, 5’-AACACGAAATAGGGCACCGT-3’, primers for hPGDS were: sense, 5’-GGGCAGAGAAAAACAAGATGT-3’, antisense, 5’-CCCCCCTAAATATGTGTCCAAG-3’. Data were analyzed as the change in cycle threshold (CT) of each cytokine transcript in comparison with β-actin. β-actin primers 5’-GAAGAGCTACGAGCTGCCTGA-3’ and 5’ -CAGACAGCACTGTGTTGGCG-3’ were purchased (Biosune Biotechnology, Shanghai, China).

### Hematoxylin-eosin staining

Nasal tissues were fixed in 4% paraformaldehyde, paraffin-embedded, and sectioned. Hematoxylin and eosin (HE) staining was performed and the eosinophil number was scored under 200× magnification in a blinded fashion. The eosinophils were counted with the final number being the average number of cells per 5 random high-power fields (hpfs).

### CRTH2 immunofluorescence staining

Immunofluorescence staining was performed as described previously (32). The paraffin sections were deparaffinized and hydrated. Heat-induced antigen retrieval was performed by heating sections in a microwave oven for 20 minutes in citrate buffer. Slides were washed and blocked using 1% bovine serum albumin and 10% goat serum for 2 hours. Sections were incubated with rabbit anti-human CRTH2 antibody (Abcam, ab150632) at 1:150 dilution at 4°C overnight and then were rinsed with PBS (phosphate buffer saline) solution and incubated with secondary Alexa fluor 647 goat anti-rabbit antibody (1:200, Abcam, ab150079) for 1 hour at room temperature in dark. Nuclei were stained with 1000μg/ml DAPI (4’, 6-diamidino-2-phenylindole) for 30 mins. The sections were sealed in antifade mounting medium. CRTH2 positive cells were scored in a blinded fashion. For the samples that were successfully stained, the slides were photographed using a confocal laser scan microscope LSM980 (Carl Zeiss, Germany) and the final number was expressed as the average number of cells per 5 random 200× hpfs.

Multiplexed immunofluorescence (mIF) assay was performed using the Opal 6-Plex Detection Kit (AKOYA #811001, USA). At room temperature, we incubated primary antibodies for major basic protein (MBP) (1:150; Boster, China), Siglec8 (1:30; Biolegend, USA), CRTH2 (1:150; Abcam, China), CD69 (1:150; Santa Cruz Biotechnology, China) for 2 hours. Following that, samples were incubated with the secondary antibody at 37°C for 10 minutes using the Opal ploymer anti-rabbit/mouse horseradish peroxidase (HRP). The Opal 6-Plex Detection Kit was subsequently used to visualize the Tyramide Signal Amplification (TSA), which contains Opal 520, Opal 570, Opal 620, and Opal 690. Then the nuclei were stained with DAPI (1:100) for 10 minutes. Image acquisition was performed with TissueFAXS (TissueGnostics).

### Flow cytometry

Peripheral blood was extracted from subjects in EDTA (ethylene diamine tetraacetic acid) tubes and then 150 μL was pipetted into respective polypropylene tubes. Negative controls only consisted of tested blood without the addition of diagnostic antibodies. ACK (Ammonium Chloride-Potassium) Lysis buffer was used to lyse red blood cells. Cells were resuspended in FACS (fluorescence-activated cell sorting) buffer at a concentration of 1 x 10^6^ cells/ml and stained with 5 μL Zombie Aqua™ Fixable Viability (Biolegend, 423101), 5 μL FITC-conjugated anti-CD16 (BioLegend, 302006), and 5 μL APC-conjugated anti-CD294 (CRTH2) (Biolegend, 350109). Finally, the cells were harvested and resuspended in 500 μL PBS. Flow cytometry was performed including live/dead viability gate with CRTH2+ eosinophils identified as SSC^high^, CD16- and CRTH2+ granulocytes. Isotype antibody controls were used to develop the gating and data analysis strategies. Flow cytometry was performed on BD FACSAria III. The data were analyzed with FlowJo.

### ELISA for PGD_2_

In brief, the nasal tissues were added to 1 mL of PBS per every 0.1 g of tissue and then homogenized on ice. After homogenization, the suspensions were centrifuged at 3,000 rpm for 10 minutes at 4°C, after which the supernatants were separated and stored at -80°C until analysis. Supernatants were assayed for the levels of PGD2 by using ELISA kits for PGD2 (Cayman Chemical, 512031) according to the manufacturer’s directions (lower limit of detection, 55 pg/mL).

### Statistical analyses

The data generated in this study were analyzed using GraphPad Prism 8 and SPSS version 26.0. Continuous data were represented as mean with standard deviation (SD) and were assessed for normality and equal variation. Chi-square test was applied for categorical variables to compare the demographic distribution and clinical variables among the different groups. One-way analysis of variance (ANOVA) was performed for comparisons of rNP, Non-rNP, and control data that passed the normality and equal variation tests, otherwise, Kruskal-Wallis test was used. The subsequent multiple comparisons for two groups were further adjusted using the Holm-Sidak’s multiple comparisons test and Dunn’s multiple comparisons test, respectively. Correlation analysis was performed using Spearman’s correlation by comparing eosinophils and SNOT-22 as a function of CRTH2 positive cell number, considering the non-normality. The ROC curve was plotted to compare the predictive ability among different variables. A P value of <0.05 was considered statistically significant.

## Results

### Demographic and clinical characteristics

**Figure 1:**
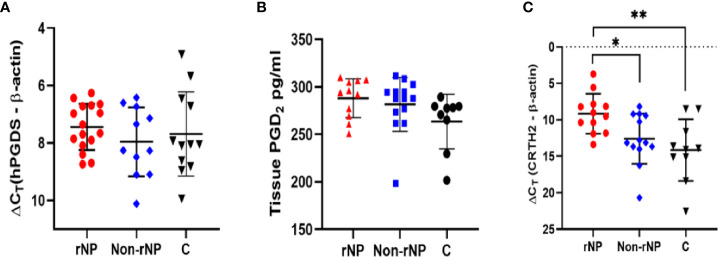
Screening of hPGDS-PGD_2_-CRTH2 pathway in nasal polyps. Nasal polyp tissues from rNP and Non-rNP groups and middle turbinate mucosa of the control group were collected and detected by real-time quantitative PCR and ELISA. No significant difference was observed among the groups in tissue hPGDS mRNA level (rNP group: n = 16; Non-rNP group: n = 11; control group: n = 12) **(A)**. The PGD2 levels of tissue homogenates measured by ELISA did not show any significant difference among these groups either (rNP group: n = 11; Non-rNP group: n = 14; control group: n = 9) **(B)**. CRTH2 mRNA level in rNP was significantly higher compared with Non-rNP and control groups (rNP group: n = 12; Non-rNP group: n = 13; control group: n = 10) **(C)**. ΔCt (the Ct value of the target gene minus the Ct value of housekeeping gene β-actin) was used to compare the data among the groups. ΔCt value was inversely proportional to the relative level of cDNA contained in the sample. C, control group. *P < 0.05, ** *P* < 0.01.

**Figure 2:**
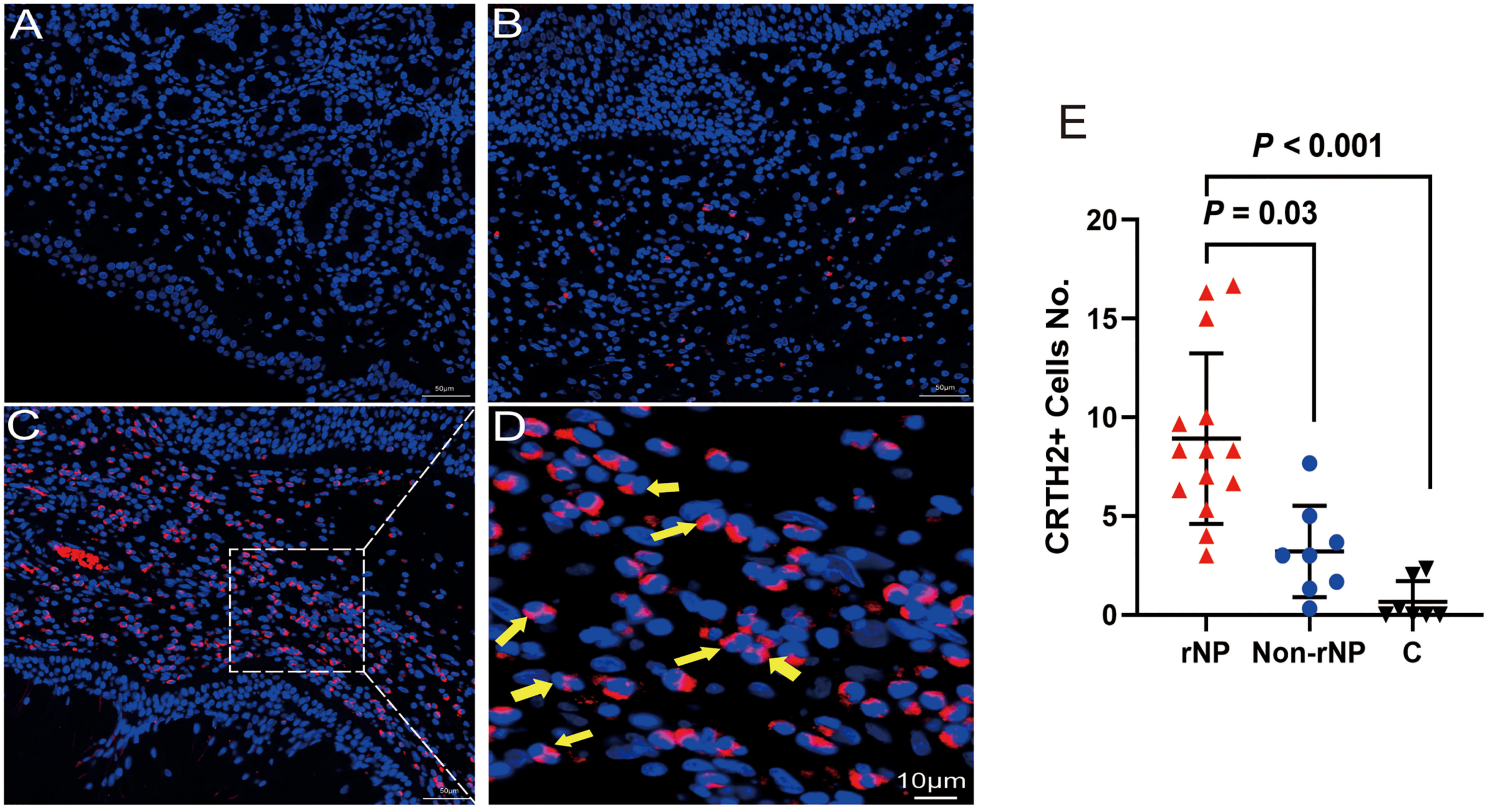
Immunofluorescence for CRTH2 (red color) in polyps from patients with NP and control tissue. **(A)** Control tissue. **(B)** Non-rNP tissue. **(C)** Tissue from a patient with rNP. **(D)** A close-up view of picture **(C)**, yellow arrows indicate eosinophils with bilobed nuclei. A dot plot showing positive CRTH2 cells/hpf for tissue samples from patients with rNP (n = 14) / Non-rNP (n = 8) / control subjects (n = 7) **(E)**. Images were generated as described in methods and analyzed by counting CRTH2+ cells with the exclusion of epithelial and glandular cells. C, control group.

Demographic and clinical characteristics of subjects are shown in **Table 1**. There were no significant differences in age, sex, allergic rhinitis, hypertension, diabetes mellitus, smoking, and drinking among the three groups. The percentage of patients with asthma was significantly higher in rNP than those in control group (*P* = 0.006). Due to the size of tissue samples, we cannot perform every experiment on all the participants. The numbers of individuals that were enrolled in each experiment were shown in each dot **Figures 1**, **2**, **4**, **5**, **7** and **Supplement Figure 2**.

**Table 1:** Characteristics of subjects enrolled in this study.

Characteristics	Recurrent nasal polyp .(n=26)	Non-recurrent nasal polyp(n=15)	Control group(n=17)	*P* value
Age, mean ± SD, y	41.7 ± 15.1	50.7 ± 9.6	37.8 ± 17.5	0.062
Female, No. (%)	8(30.7)	5(33.3)	6(35.2)	0.952
Asthma, No. (%)	11(42.3)	3(20.0)	0(0.0)	0.006
Allergic rhinitis, No. (%)	9(34.6)	2(13.3)	3(17.6)	0.234
Hypertension, No. (%)	6(23.0)	2(13.3)	4(23.5)	0.716
Diabetes mellitus, No. (%)	1(3.8)	1 (6.6)	0(0.0)	0.581
Smoking, No. (%)	6(23.0)	5(33.3)	2(11.7)	0.342
^†^Drinking, No. (%)	11(42.3)	7(46.6)	3(17.6)	0.160
Blood eosinophil count (10^9^/L), mean ± SD	0.48 ± 0.07	0.24 ± 0.03	0.14 ± 0.26	<0.001
Lund-Mackay score, mean ± SD	16.6 ± 5.7	13.5 ± 5.6	–	0.118

SD, standard deviation; No, number.

^†^A regular drinker was defined as a person who drank alcoholic beverages at least once a month.

### Screening of hPGDS-PGD_2_-CRTH2 pathway in nasal polyps

To explore the role that hPGDS-PGD_2_-CRTH2 pathway plays in CRSwNP, especially in the recurrence of nasal polyps, we screened the expression of hPGDS-PGD_2_-CRTH2 in nasal polyps defined by their tendency to recur post-surgery. No significant differences were observed among these groups in tissue hPGDS mRNA levels (**Figure 1A**) and PGD_2_ levels of tissue homogenates measured by ELISA (**Figure 1B**). In contrast, CRTH2 mRNA level in rNP group was significantly higher compared with those in Non-rNP (**Figure 1C**, *P* = 0.04) and control groups (**Figure 1C**, *P* = 0.006). Taken together, these data suggest a prominent role of CRTH2 in the hPGDS-PGD_2_-CRTH2 pathway of rNP.

### Immunofluorescence staining of CRTH2 in nasal polyps

**Figure 3:**
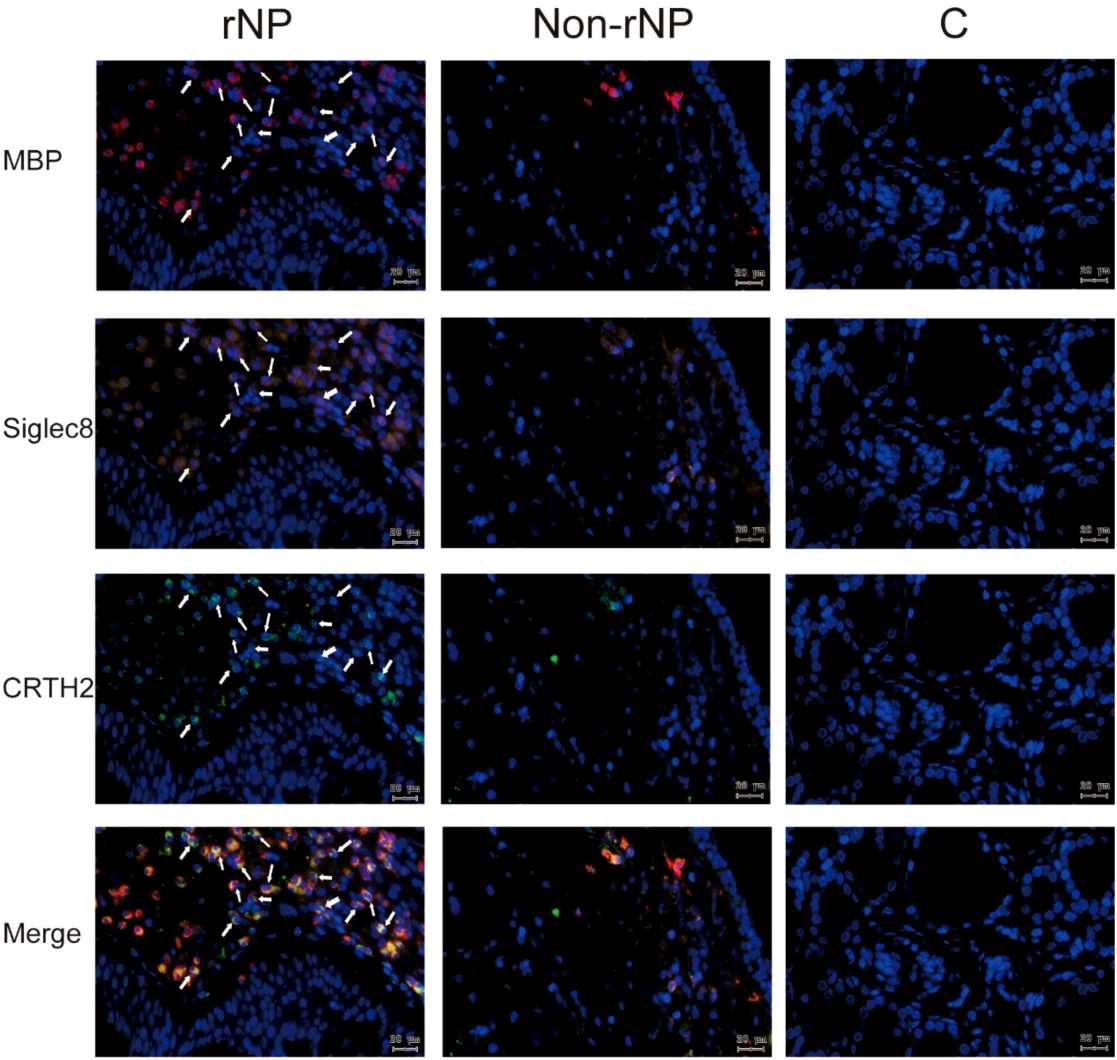
Multiplex immunofluorescence staining of nasal tissues for MBP (red), Siglec8 (orange), CRTH2 (green), and DAPI (blue) were performed. Representative fluorescence images of rNP group, Non-rNP group, and control group. White Arrows indicated eosinophils that expressed the corresponding markers. C, control group.

To detect the level of CRTH2 protein and define the cellular source, we performed immunofluorescence staining on nasal tissue samples. Representative images were shown in **Figure 2**, and the isotype control images were displayed in **Supplement Figure 1**. Essentially no CRTH2 staining was observed in control tissue (**Figure 2A**), moderate CRTH2 levels were detected in Non-rNP samples (**Figure 2B**), and the highest levels were found in rNP (**Figure 2C**). As shown in **Figure 2D**, most of these cells in rNP were supposed to be eosinophils by typical bilobed nuclei (yellow arrows). To further explore the expression of CRTH2 in nasal polyps, the number of CRTH2+ cells per hpf in immunofluorescence staining was determined. The rNP group presented with more CRTH2+ cells compared with Non-rNP (**Figure 2E**, *P* = 0.03) and control group (**Figure 2E**, *P* < 0.001). These results further support our mRNA data showing high expression of CRTH2 in rNP. In order to confirm the CRTH2 expression on eosinophils, Siglec8 and MBP were used as eosinophil markers in multiplexed immunofluorescence staining (**Figure 3** **)** on these tissues, which showed that most of the CRTH2 positive cells were co-stained with Siglec8 and MBP, especially in the rNP group. Overall, these data indicated the higher expression of CRTH2 in rNP and the close relationship between CRTH2 and eosinophils.

### High CRTH2 expression in rNP is not driven by concomitant asthma

**Figure 4:**
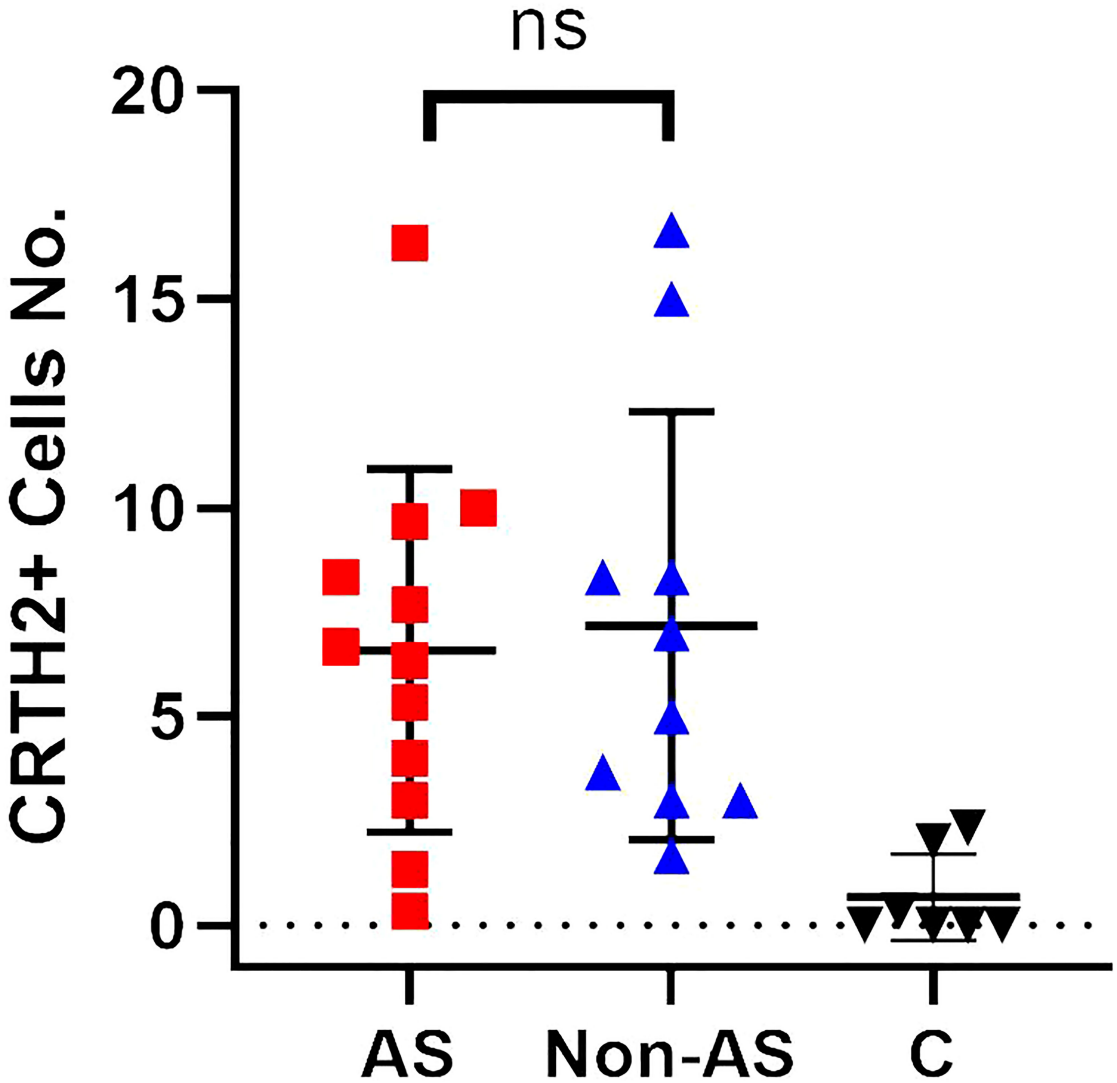
A dot plot showing positive CRTH2 cells/hpf for tissue samples from nasal polyps with concomitant asthma (AS) (n = 12) / nasal polyps without concomitant asthma (Non-AS) (n = 10) / control subjects (n = 7). ns, not significant.

As more recurrent NP subjects had concomitant asthma (**Table 1**) and previous research had reported the higher expression of CRTH2 in asthma (33), to further address whether the CRTH2 difference between rNP and Non-rNP was driven by asthma, we analyzed the expression of CRTH2 in nasal polyps obtained from asthmatic and non-asthmatic subjects. However, no significant difference in CRTH2 expression was found between the asthmatic and non-asthmatic groups (**Figure 4**), demonstrating that higher CRTH2 expression may be an independent feature of rNP and that this is not being driven by the presence of asthma.

### Over-expression of CRTH2 is associated with eosinophilic inflammation in NP

**Figure 5:**
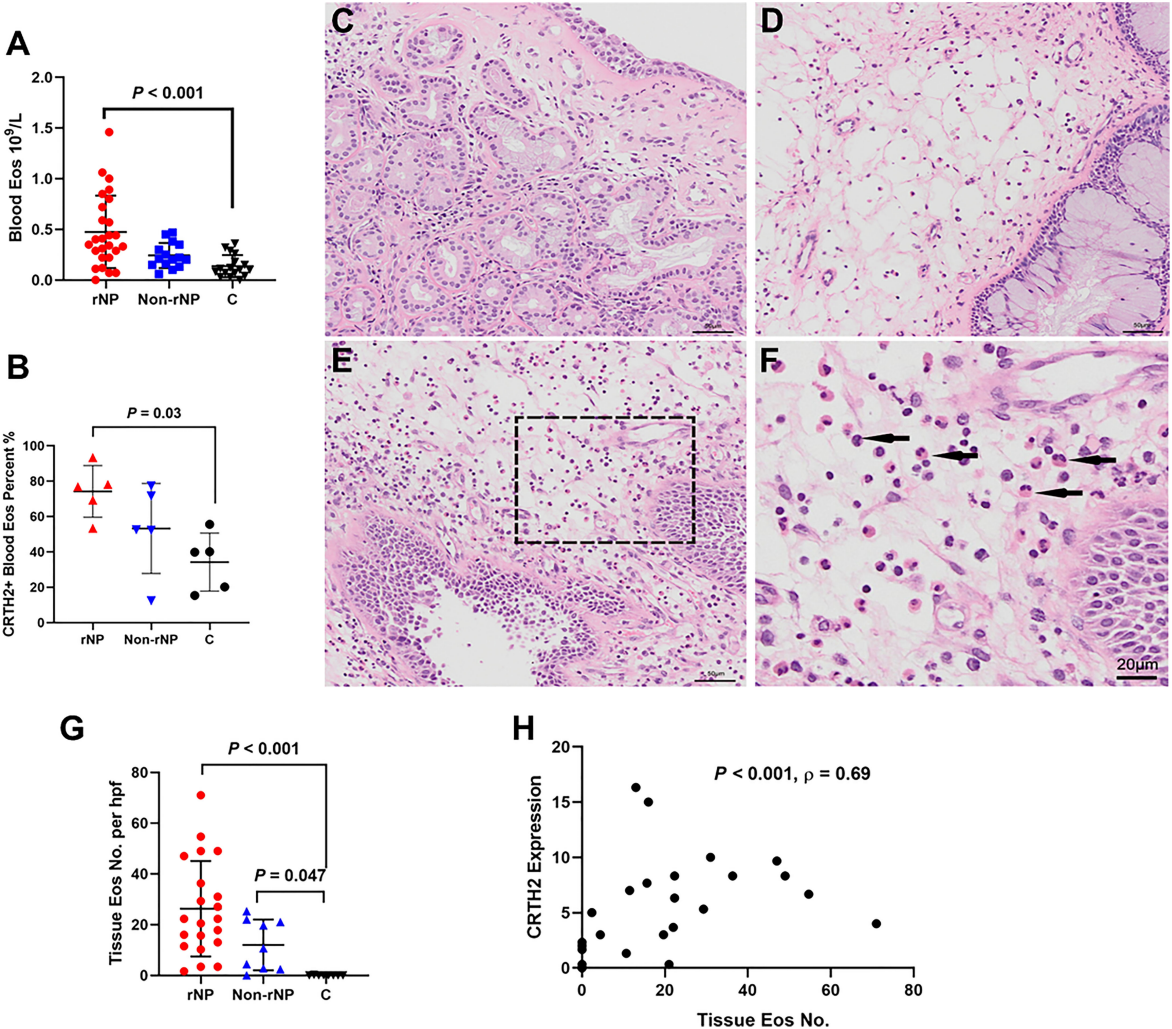
Over-expression of CRTH2 is associated with eosinophil inflammation in NP. **(A)** The blood eosinophil count in patients with rNP was significantly higher than that in the control group, while the difference between control and Non-rNP was not significant (rNP group: n = 26; Non-rNP group: n = 15; control group: n = 17). **(B)** Eosinophils of the rNP group presented higher CRTH2 expression than that of controls as shown by the higher percentage of CRTH2+ in blood eosinophils detected by flow cytometry (n = 5 per group). **(C)** HE staining of middle turbinate mucosa of the control group, **(D)** Non-rNP, **(E)** rNP, and **(F)** a closer view of picture **(E)**, with the black arrows indicating eosinophils. **(G)** Tissue eosinophils were further scored as mean number of eosinophils/200×hpf in HE stained sections. rNP (n = 21) and Non-rNP (n = 9) displayed significantly higher eosinophil numbers compared with controls (n = 8). **(H)** Correlation between eosinophils number and CRTH2+ expression detected by immunofluorescence in nasal tissues (n = 27). C, control group.

As eosinophils were identified as being the most numerous cell expressing CRTH2 in nasal polyps, we further explored the association between CRTH2 and eosinophils in nasal polyps. It can be seen in **Figure 5A** that the circulating eosinophil count in the rNP group was significantly higher than that in the control group (*P* < 0.001). The counts of blood neutrophils, monocytes, and lymphocytes were also analyzed, but no significant differences were observed among these groups (**Supplement Figure 2**). To evaluate CRTH2 receptor expression on blood eosinophils, flow cytometry was performed and revealed a higher CRTH2 positive percentage in total eosinophils in patients with rNP compared with that in the control group (**Figure 5B**, *P* = 0.03), suggesting the blood eosinophils of rNP are more active with higher CRTH2 expression even on a per-cell basis. Gating strategy, isotype controls, and representative images are displayed in **Supplement Figure 3**.

**Figure 6:**
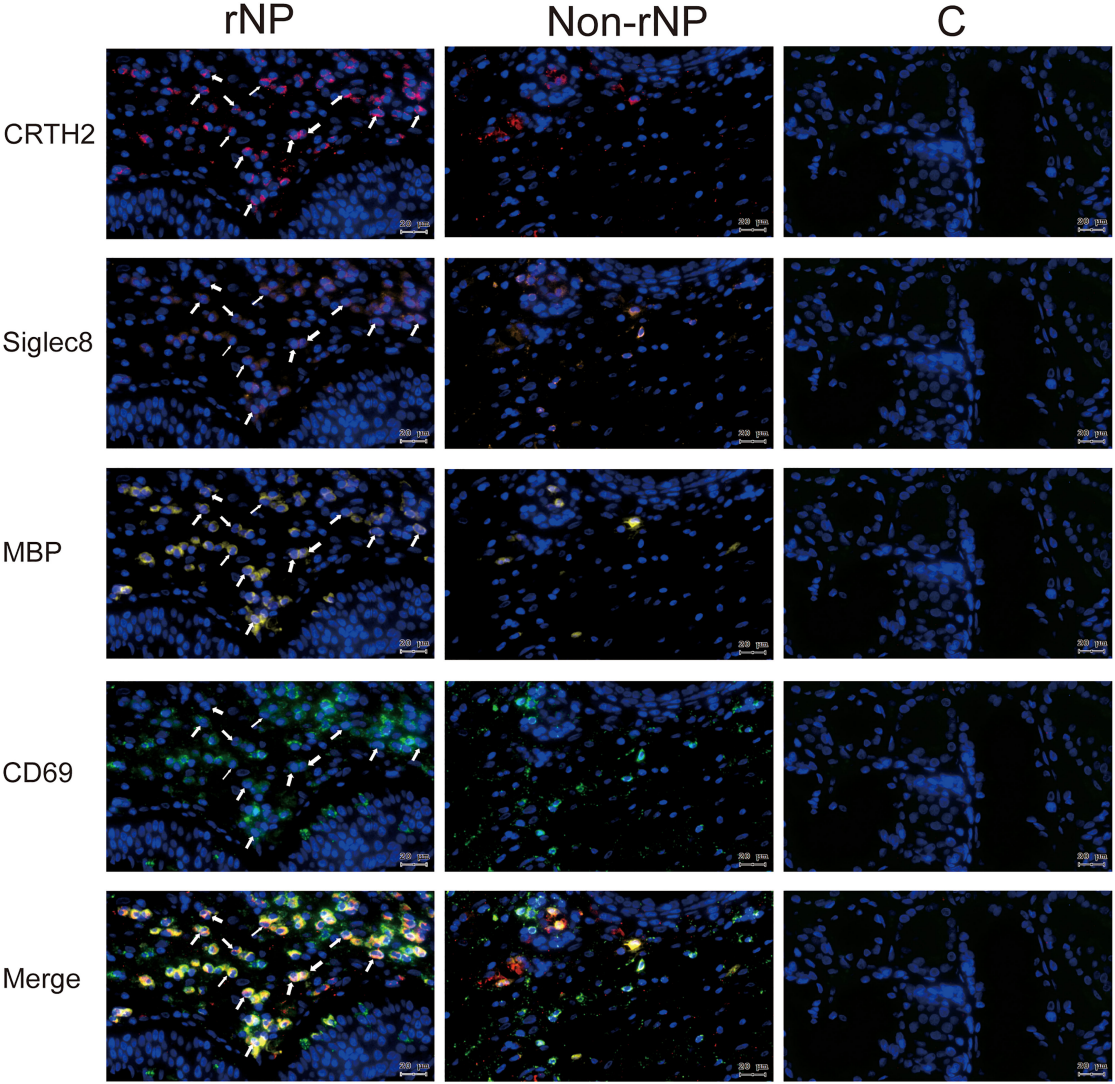
Representative tissue images stained using multiplex immunofluorescence staining for CRTH2 (red), Siglec8 (orange), MBP (yellow), CD69 (green), and DAPI (blue) in rNP group, Non-rNP group, and control group. White Arrows indicated eosinophils that expressed the corresponding markers. C, control group.

To evaluate tissue eosinophils, HE staining was performed on nasal tissues. A prominent expression of eosinophils was observed in rNP, while almost no eosinophils were detected in control tissue (**Figures 5C–F**), with the tissue eosinophil number in the rNP and Non-rNP group significantly higher than that in the control group (**Figure 5G**, *P*<0.001 and *P*=0.047, respectively). Further analysis demonstrated tissue CRTH2+ expression positively correlated with tissue eosinophil number (**Figure 5H**, Spearman’s ρ=0.69, *P* < 0.001). Additionally, to identify the characteristics of the CRTH2+ eosinophils, we also performed multiplexed immunofluorescence staining with CD69, a marker of eosinophil activation. Extensive co-stainings were observed among CD69, CRTH2, MBP, and Siglec8 in rNP tissues, suggesting an activated state of the CRTH2+ eosinophil in rNP, a phenomenon that was not shown in the control tissues (**Figure 6**). Taken together, these results indicate the expression of CRTH2 is closely associated with eosinophilic inflammation in NP patients.

### Clinical implications of CRTH2 expression in patients with CRSwNP

**Figure 7:**
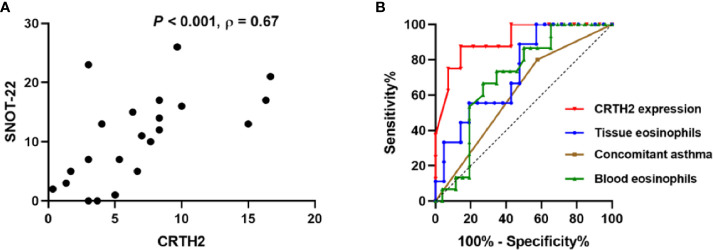
**(A)** Correlation between CRTH2 and SNOT-22 score 12-months postoperatively (n = 22). **(B)** Receiver operating characteristic curves of tissue CRTH2 expression (red line, AUC=0.9107, *P*=0.002, 95% CI: 0.7855 to 1.000), tissue eosinophil number (blue, AUC= 0.7354, *P*=0.044, 95% CI: 0.5497 to 0.9212), concomitant asthma (brown, AUC=0.6115, *P*=0.239, 95% CI: 0.4348 to 0.7883) and blood eosinophil number (green, AUC=0.7083, *P*=0.031, 95% CI: 0.5435 to 0.8642), respectively. AUC, area under the receiver operating characteristic curve.

The over-expression of CRTH2 in the rNP group and its close association with eosinophilic inflammation prompted us to query whether its expression affected the symptoms and prognosis of CRSwNP. Spearman’s analysis revealed a positive correlation between the CRTH2 expression and the 12-month postoperative SNOT-22 score (**Figure 7A**, Spearman’s ρ=0.67, *P*<0.001). The preoperative SNOT-22 score and Lund-Mackay score were also assessed, but no significant associations were detected between these data and CRTH2 expression (data not shown). Together these data suggest that higher CRTH2 expression may indicate a poor postoperative prognosis of NP.

To further address the factors predicting the recurrence of nasal polyps, ROC curves were performed. CRTH2 expression showed the highest accuracy as a predictor for rNP (**Figure 7B**, AUC=0.9107) compared with tissue eosinophil number (AUC=0.7354), concomitant asthma (AUC=0.6115), and blood eosinophil number (AUC=0.7083). In this regard, when we set a cutoff point value of >5 for CRTH2 expression, the sensitivity and specificity for predicting rNP were 87.50% and 85.71%, respectively, an accuracy that could not be achieved by either eosinophil number or concomitant asthma.

## Discussion

Eosinophilic CRSwNP is usually characterized by recurrent nasal polyp growth following surgical removal. Even in Asian populations, in which non-eosinophilic or neutrophilic polyps are the dominant type (34), a large number of tissue eosinophils can still be used as a reliable indicator for predicting nasal polyp recurrence (35). Consistent with these findings, we found that the circulating eosinophil number, while not that of other inflammatory cells (neutrophils, monocytes, and lymphocytes), was significantly higher in the rNP group than that in the control group (**Figure 5A** and **Supplement Figure 2**), and a more prominent difference in tissue eosinophils number was also observed between the rNP group and control group (**Figure 5G**). These observations support the important role that eosinophils play in rNP. The potential reason may be that eosinophils contribute to enhanced inflammation, tissue remodeling, a loss of matrix deposition, and an increase in tissue edema (36). Thus, understanding the factors that promote eosinophil migration and accumulation in the tissue is important for preventing nasal polyp recurrence.

PGD_2_ can activate Th2 lymphocytes, eosinophils, basophils, and ILC2s, inducing cytokine production and enhancing chemotaxis through CRTH2 receptor engagement (37, 38), and thus play an important role in asthma and allergic diseases. The hPGDS-PGD_2_-CRTH2 pathway was up-regulated in patients with severe, poorly controlled asthma (33). CRTH2 expression on leukocytes in allergic nasal mucosa was significantly up-regulated compared with those in nonallergic nasal mucosa (17). However, the exploration of CRTH2 in nasal polyps is still limited and debated. Yamamoto et al. demonstrated a down-regulation of CRTH2 in NP compared to uncinate process mucosae (24), but Nantel et al. reported CRTH2 was only detected in the nasal mucosa of subjects suffering from polyposis, while not in normal mucosa (25). Our previous research demonstrated over-expression of hPGDS-PGD_2_ in eosinophils of AERD patients (14). Here, we further screened the hPGDS-PGD_2_-CRTH2 pathway in nasal polyps and confirmed the over-expression of CRTH2 in NP, specifically in the setting of rNP. Interestingly, no significant differences were observed among rNP group, Non-rNP, and control groups in tissue hPGDS mRNA levels and PGD_2_ levels of tissue homogenates in accordance with the findings of Nordström et al. which was based on nasal secretions (39). However, the detection by immunofluorescence staining confirmed higher CRTH2 expression in rNP compared to Non-rNP, suggesting a greater role of CRTH2 in the hPGDS-PGD_2_-CRTH2 pathway driving rNP. Consistent with the study by Yamamoto and his colleagues (24), we also found that CRTH2 was selectively expressed in inflammatory cells, but they demonstrated a lower CRTH2 mRNA expression in NP, which is opposite to our research. This difference may be led by the different control tissues used in the two studies, as we chose middle turbinates of patients with deviated nasal septum as control, while Yamamoto et al. used uncinate process mucosae of CRS patients, which may be inflammatory mucosae. Further exploration is needed to elucidate these debates.

Of note, although more recurrent NP subjects had concomitant asthma (**Table 1**), CRTH2 expression was not greater in NPs derived from asthmatics, indicating that the over-expression of CRTH2 in nasal polyps is a specific feature of rNP independent of asthma status.

In asthma and allergic rhinitis, CRTH2 is essential for sustained eosinophilic inflammation (40). However, the relationship between CRTH2 and eosinophil inflammation in nasal polyps has not been reported. Here we demonstrated the close relationship between CRTH2 and eosinophilic inflammation in nasal polyps (**Figure 5H**). Moreover, our data also revealed the over-expression of CRTH2 on blood eosinophils of patients with rNP (**Figure 5B**), of interest, this over-expression in blood was not as prominent as that in tissue, arguably reflecting that the high-expressing CRTH2+ eosinophils had selectively migrated into tissue. In agreement with the findings of Miki-Hosokawa et al. the increased CD69 expression on activated eosinophils in rNP was also been found (41). Together these observations argue that the over-expression of CRTH2 may contribute to the migration and accumulation of eosinophils in rNP through enhancing the sensitivity of eosinophils to PGD_2_, and thus contribute to this recurrence of nasal polyps. On the other hand, Th2 lymphocytes and ILC2s are also linked with tissue eosinophil accumulation and have a potential role in the activation and survival of eosinophils during the type 2 immune response (42). As such, we cannot exclude the impact of other inflammatory cells such as Th2 and ILC2 on NP recurrence. Further exploration of the relationship between CRTH2 and ILC2/Th2 and other cell types in rNP is warranted.

Eosinophilia and concomitant asthma were reported to be the risk factors of nasal polyps recurrence (5–8). In this research, we further demonstrated that up-regulated CRTH2 expression was linked with poor prognosis and, importantly, that CRTH2 expression was a better predictor for nasal polyp recurrence compared with either eosinophil expression or asthma diagnosis when analyzed by ROC. As eosinophils are not the only source of CRTH2-expressing cells, higher CRTH2 expression may also indicate higher numbers of activated Th2 cells, ILC2, and other inflammatory cells in rNP, which will aggravate inflammation, promote nasal polyp recurrence and contribute to the poor prognosis. As such, the expression of CRTH2 may be a good marker for all these inflammatory cells while not just eosinophils. Moreover, we also confirmed the significant difference between rNP and Non-rNP in tissue CRTH2 expression (**Figure 2E**) while not in tissue (**Figure 5G**) and blood eosinophils number (**Figure 5A**) in this study, indicating CRTH2 plays a more prominent role in NP recurrence than eosinophils. Collectively, these data may explain the better accuracy of CRTH2 expression in predicting NP recurrence and poor prognosis in comparison to that predicted by tissue eosinophils alone.

Taken together, these findings reminded us that whether CRTH2 antagonism could be a potential treatment option for eosinophilic CRSwNP and rNP, especially in those subjects displaying the highest level of this receptor. Several CRTH2 antagonists have been developed and tested in clinical trials of asthma and allergic diseases, but their clinical benefits still need to be determined (18, 19). CRTH2 antagonists Setipiprant (43), BI671800 (44), and OC000459 (45) were proved to be effective in treating allergic rhinitis symptoms. The CRTH2 antagonist fevipiprant safely improved asthma outcomes compared to placebo, but most of the differences did not reach the minimal clinically important difference (20–22, 46). To date, only one phase 3b study (NCT03681093) evaluated fevipiprant, as an add-on to nasal spray standard-of-care in reducing NP size in patients with nasal polyposis and concomitant asthma, but no prominent clinical benefits were observed, which may be explained by our study that NP with concomitant asthma did not present an over-expression of CRTH2 (47). Given the heterogenous responses to CRTH2 antagonists with some trials failing to produce clinically important differences (22), there is a need to identify the most responsive NP endotype likely to respond to CRTH2 antagonism. The up-regulated expression of CRTH2 in rNP and the significant positive correlation between CRTH2 and post-operative SNOT-22 in our results strongly suggest that recurrent CRSwNP may be one such subtype likely to benefit from CRTH2 antagonism which required to be further confirmed *in vivo* trails. Future studies that specifically recruit subjects displaying high levels of CRTH2, such as those with AERD or rNP, may be needed.

There are several limitations to this study. The linkage of CRTH2 to the underlying biology is complicated in CRSwNP, as besides eosinophils, Th2, ILC2, and other inflammatory cells are also likely to be the source of CRTH2 over-expression and thus contribute to the recurrence of NP through this receptor, we cannot exclude the effects of these cells. *In vitro* experiments with purified eosinophils from rNP patients may be needed to determine the exact role that CRTH2 plays in eosinophil migration and accumulation. Second, PGD2 is also synthesized in necrotic tissues and cell lysates, therefore, the supernatants of tissue homogenates might not accurately reflect the physiological state of these tissues, further detection of PGD2 with nasal wash fluid or supernatants of the fresh tissues that were collected after washing the resting cells and tissue pieces may be needed. Finally, this study was performed just in the Chinese population, which may limit the external validity of this research, further studies with more samples and diverse races may be needed to validate the generalizability of this research.

## Conclusion

This study demonstrated the over-expression of CRTH2 in rNP, a feature that is independent of concomitant asthma. Together with the findings that CRTH2 expression correlated with the extent of eosinophilic inflammation and postoperative SNOT-22 score, we may anticipate that in patients with the over-expressing phenotype, a CRTH2 antagonist may be a potential therapeutic option in patients with rNP that have proven to be unresponsive to standard therapy.

## Data availability statement

The raw data supporting the conclusions of this article will be made available by the authors, without undue reservation.

## Ethics statement

The studies involving human participants were reviewed and approved by The Medical Ethics Committee of Qilu Hospital of Shandong University. Written informed consent to participate in this study was provided by the participants or the participants’ legal guardian/next of kin.

## Author contributions

XF designed the study. WC, SH and XF drafted the manuscript. WC, SH, XX, PY, XF, CD and XL contributed to the enrollment of subjects and data collection. WC and SH performed the experiments. XY, SH and WC performed the statistical analysis. XF, XY, LB and ML contributed to interpretation of the results, reviewed and edited the manuscript. All authors contributed to the article and approved the submitted version.

## Funding

XF is supported by the National Natural Science Foundation of China (81700890, 82171106), Taishan Scholar Program of Shandong Province (tsqn202103166). CD is supported by Shandong Natural Science Foundation (ZR2020QH151). LB is supported by NIH UO1 AI123337, R21 AI151496, and R21 AI151497.

## Conflict of interest

The authors declare that the research was conducted in the absence of any commercial or financial relationships that could be construed as a potential conflict of interest.

## Publisher’s note

All claims expressed in this article are solely those of the authors and do not necessarily represent those of their affiliated organizations, or those of the publisher, the editors and the reviewers. Any product that may be evaluated in this article, or claim that may be made by its manufacturer, is not guaranteed or endorsed by the publisher.

## Abbreviations

CRSwNP, chronic rhinosinusitis with nasal polyps; NP, nasal polyp; CRTH2, chemoattractant receptor-homologous molecule expressed on Th2 cells; ROC, receiver operating characteristic curves; CRS, chronic rhinosinusitis; PGD_2_, prostaglandin D2; Th2, type 2 helper T lymphocytes; ILC2, group 2 innate lymphoid cells; hPGDS, hematopoietic prostaglandin D2 synthase; AERD, aspirin-exacerbated respiratory disease; rNP, recurrent nasal polyps; SNOT-22, the sino-nasal outcome test-22; HE, hematoxylin and eosin; hpfs, high-power fields; EDTA, ethylene diamine tetraacetic acid; ELISA, enzyme-linked immunoassay; AUC, area under the curve.
