# Supplementary material for: Over-expression of CRTH2 indicates eosinophilic inflammation and poor prognosis in recurrent nasal polyps
Source: Front Immunol. 2022 Nov 18;13:1046426. doi: 10.3389/fimmu.2022.1046426 (PMC9716025; doi:10.3389/fimmu.2022.1046426)
Supplement: Supplementary file 1 [file DataSheet_1.pdf]

Supplementary Material

Figure legends:

Supplement Figure 1. Isotype control of immunofluorescence for CRTH2. **(A)** Tissue was incubated with polyclonal rabbit sera and allophycocyanin-labeled secondary antibody. **(B)** Tissue was incubated with rabbit anti-CRTH2 primary and allophycocyanin-labeled secondary antibodies.

Supplement Figure 2: The number of blood neutrophils **(A)**, monocytes **(B)**, and lymphocytes **(C)** in patients with rNP, Non-rNP, and control group.

Supplement Figure 3: Gating strategy in blood. **(A)** Peripheral blood was extracted and ACK Lysis buffer was used to lyse red blood cells, then CRTH2-positive eosinophils were identified as SCC^{high}, FSC^{high}, live, CD16- and CRTH2+ granulocytes. **(B)** Isotype control staining demonstrated a lack of non-specific staining. **(C)** Representative images of CRTH2+ eosinophils in rNP, Non-rNP, and control groups were displayed.

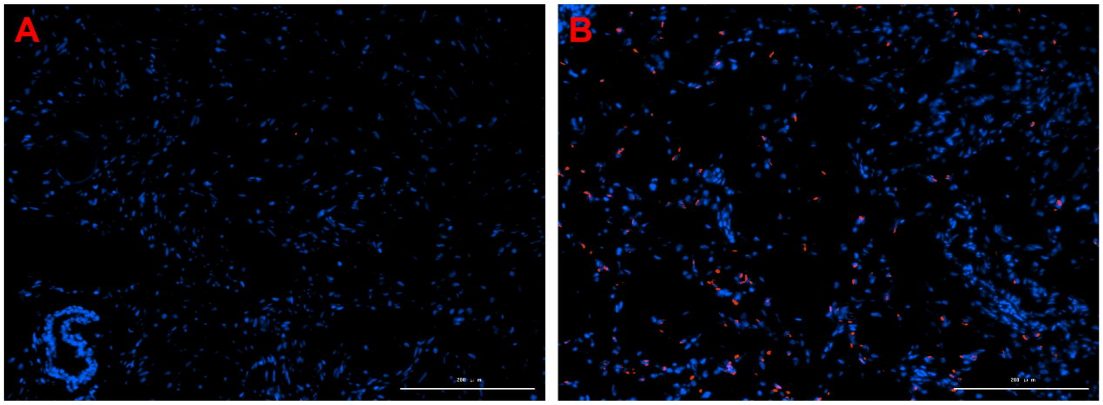

Supplement Figure 1. Isotype control of immunofluorescence for CRTH2. **(A)** Tissue was incubated with polyclonal rabbit sera and allophycocyanin-labeled secondary antibody. **(B)** Tissue was incubated with rabbit anti-CRTH2 primary and allophycocyanin-labeled secondary antibodies.

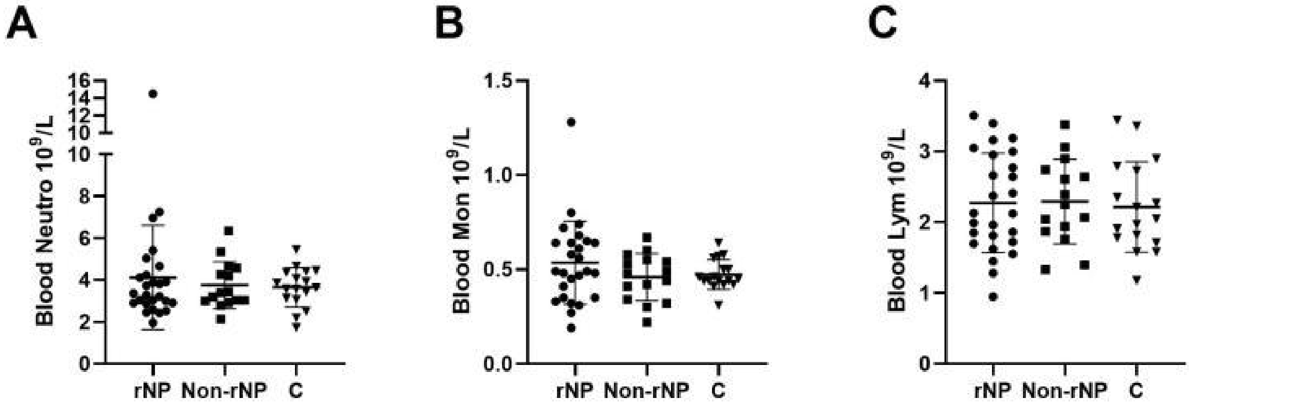

Supplement Figure 2: The number of blood neutrophils **(A)**, monocytes **(B)**, and lymphocytes **(C)** in patients with rNP, Non-rNP, and control group.

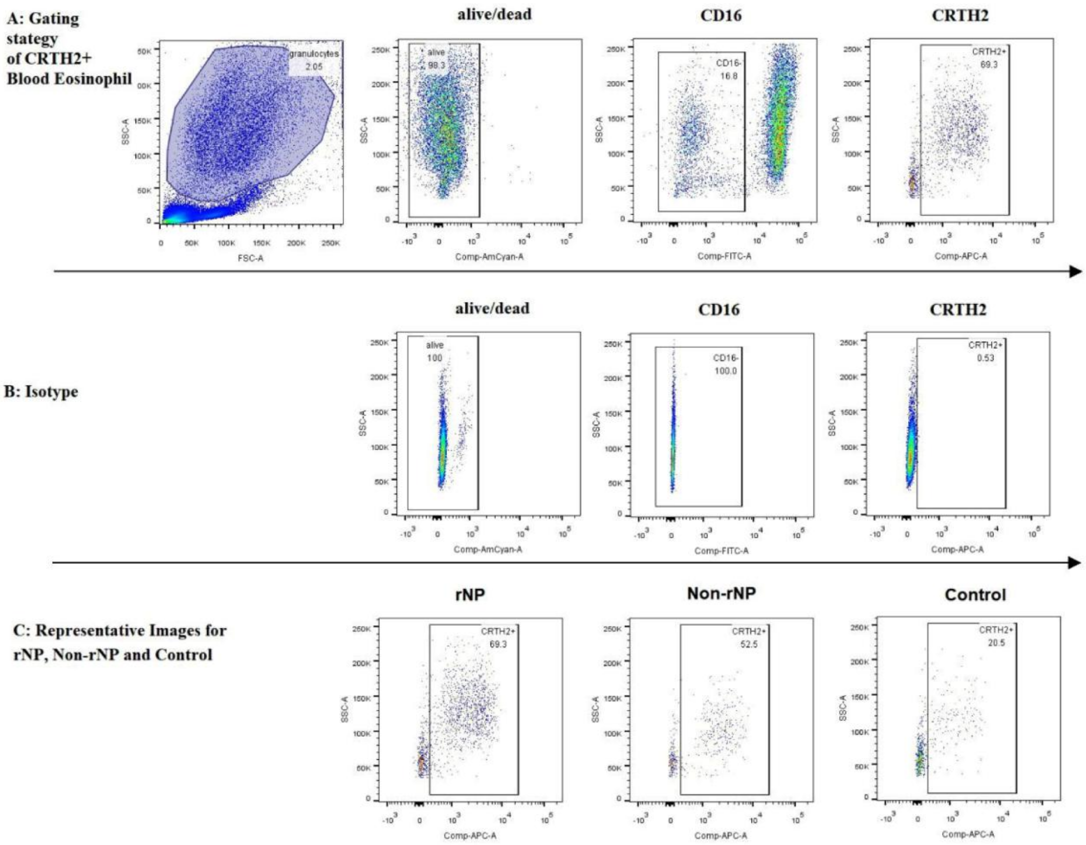

Supplement Figure 3: Gating strategy in blood. **(A)** Peripheral blood was extracted and ACK Lysis buffer was used to lyse red blood cells, then CRTH2-positive eosinophils were identified as SCC^{high}, FSC^{high}, live, CD16- and CRTH2+ granulocytes. **(B)** Isotype control staining demonstrated a lack of non-specific staining. **(C)** Representative images of CRTH2+ eosinophils in rNP, Non-rNP, and control groups were displayed.
